# Prolyl-Isomerase Pin1 Controls Key fMLP-Induced Neutrophil Functions

**DOI:** 10.3390/biomedicines9091130

**Published:** 2021-09-01

**Authors:** Samia Bedouhene, Min Liu, Nassima Senani, Tarek Boussetta, Coralie Pintard, Pham My-Chan Dang, Jamel El-Benna

**Affiliations:** 1 Centre de Recherche sur l’Inflammation, Laboratoire d’Excellence Inflamex, Faculté de Médecine Xavier Bichat, Université de Paris INSERM U1149, CNRS ERL 8252, 75018 Paris, France; bed_samia@yahoo.fr (S.B.); min.liu@ythbt.com (M.L.); boussettatarek@gmail.com (T.B.); coralie.pintard@inserm.fr (C.P.); my-chan.dang@inserm.fr (P.M.-C.D.); 2 Laboratoire de Biochimie Appliquée et de Biotechnologie, Faculté des Sciences Biologiques et des Sciences Agronomiques, Université Mouloud Mammeri, BPN 17, Tizi-Ouzou 15000, Algeria; senanibiochem@yahoo.fr; 3 College of Chemical Engineering, Nanjing Forestry University, Nanjing 210037, China; 4 Yitong Food Industry Co., Ltd., Xuzhou 221000, China

**Keywords:** neutrophil, prolyl-isomerase, Pin1, PiB, juglone, fMLP, chemotaxis, degranulation, respiratory burst, inflammation

## Abstract

Neutrophils are key cells of the innate immune and inflammatory responses. They are the first blood cells to migrate to the infection site where they release high amounts of reactive oxygen species (ROS) and several peptides and enzymes required for microbial killing. However, excessive neutrophil activation can induce tissue injury participating in inflammation, thus the characterization of the enzymes involved in neutrophil activation could help to identify new pharmacological targets to treat inflammation. The prolyl-isomerase Pin1 is a ubiquitous enzyme involved in several functions, however, its role in neutrophil functions is less known. In this study, we show that the bacterial peptide N-formyl-methionyl-leucyl-phenylalanine (fMLP or fMLF), a G-protein coupled receptor (GPCR) agonist-induced Pin1 activation in human neutrophils. PiB and juglone, two Pin1 inhibitors inhibited Pin1 activity in neutrophils and consequently inhibited fMLP-induced chemotaxis and -degranulation of azurophil and specific granules as measured by myeloperoxidase and neutrophil gelatinase-associated lipocalin (NGAL) release respectively. We also showed that PiB inhibited TNFα + fMLP-induced superoxide production, confirming the effect of juglone. These data show that inhibitors of Pin1 impaired key pro-inflammatory neutrophil functions elicited by GPCR activation and suggest that Pin1 could control neutrophil inflammatory functions.

## 1. Introduction

Neutrophils constitute more than 60% of circulating leukocytes in humans. They play a key role in host defense against invading microbes [1,2]. Neutrophils are in a resting state in the blood, they are the first cells to migrate out of the blood to reach the infection site. The oriented migration of neutrophils towards the infection site also called chemotaxis is an essential function that allows them to reach and kill microbes [3]. Neutrophils can be attracted by the bacterial peptide *N*-formyl-methionyl-leucyl-phenylalanine (fMLP), Interleukin 8 (IL-8), the fifth complement fragment (C5a), the Platelet Activating Factor (PAF) and leukotriene B4 (LTB4) [3,4].

At the infectious site, neutrophils recognize the microbe via different receptors and ligands, and recognition is generally followed by phagocytosis which triggers neutrophil killing mechanisms that involve mainly the release of toxic molecules from granules and from oxygen metabolism called a respiratory burst [5,6,7]. Neutrophils contain four types of granules or vesicles that have different compositions and density which are called azurophil granules, specific granules, gelatinase-rich granules, and the highly mobilizable secretory vesicles [7,8]. Azurophil granules (or primary granules) contain mainly myeloperoxidase (MPO), elastase, PR3, and cathepsins. Specific granules (or secondary granules) contain mainly lactoferrin, neutrophil gelatinase-associated lipocalin (NGAL) membrane receptors, and cytochrome b558. Gelatinase-rich granules (or tertiary granules), essentially contain gelatinase and membrane receptors, and the highly mobilizable secretory vesicles contain plasma proteins. The release of these granules, also called degranulation, is important for immunity and inflammation [7,8]. The respiratory burst is mediated by the phagocyte NADPH oxidase (NOX2), which produces superoxide anion (O_2_°−) the precursor of other ROS such as hydrogen peroxide (H_2_O_2_), which is used by myeloperoxidase to produce hypochloric acid (HOCl), a very harmful agent [9,10,11]. While these neutrophil functions are required for host defense, their excessive recruitment and activation can induce tissue injury contributing to enhanced inflammatory reaction involved in several diseases such as rheumatoid arthritis, inflammatory bowel diseases, lung inflammatory diseases, septic shock [2,9,11]. Thus, understanding how these functions are controlled could help to develop new approaches to inhibit neutrophil functions when they are up-regulated.

Pin1 is a peptidyl-prolyl “cis-trans” isomerase (PPIase), which isomerizes specifically phosphorylated-Serine/Threonine-Proline motifs [12,13]. Pin1 is an 18 kDa protein composed of two conserved domains, the N-terminal WW domain serves to recognize and bind specific phospho-proteins, and the C-terminal domain catalyzes the isomerization reaction [14,15]. Pin1 is ubiquitously expressed and plays an important role in cell functions such as cell cycle, apoptosis, protein synthesis, and protein degradation [16,17,18]. Pin1 is also involved in numerous diseases, such as Alzheimer’s disease, ageing, metabolic syndromes, and cancers [18,19,20]. Indeed, Pin1 is known to bind to several phosphorylated proteins and to induce their *cis-trans* isomerization and conformational changes. Regarding neutrophils, Pin1 is expressed in human neutrophils and mediates NADPH oxidase hyper-activation or priming induced by TNFα [21], CL097 (a TLR7/8 agonist) [22,23], and LPS (a TLR4 agonist) [24]. Pin1 is contuitively active in human neutrophils and its activity can be enhanced by TNFα [21], CL097 [22,23], and LPS [24]. Pin1 binds to phosphorylated Ser345 of p47phox and induces a conformational change of the protein to facilitate its phosphorylation by PKC and its binding to p22phox and NADPH oxidase assembly and activation [21]. However, the implication of Pin1 in other neutrophil functions is less documented.

**Figure 1:**
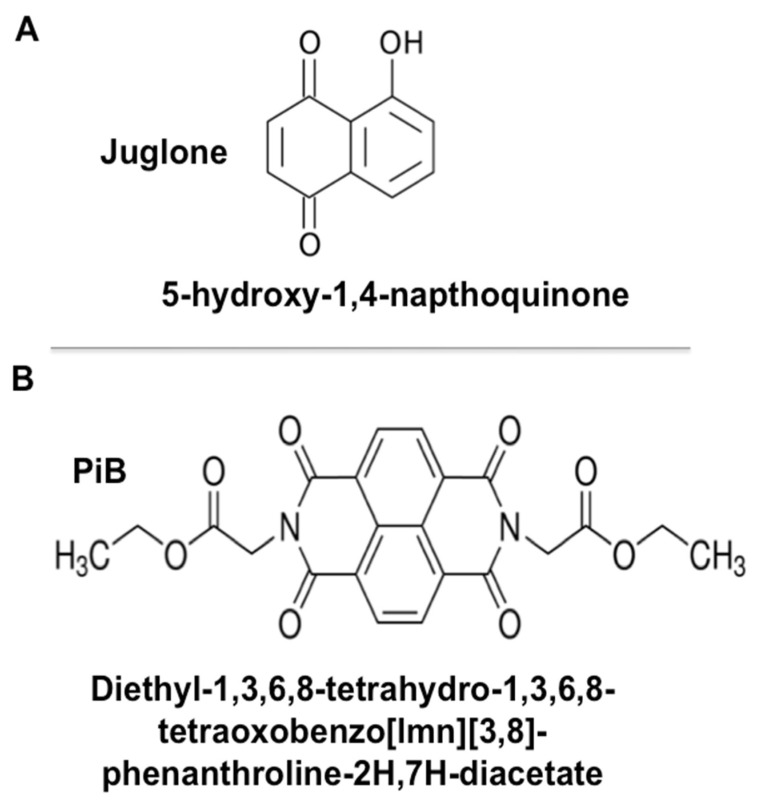
Molecular structure of juglone and PiB. (**A**) Juglone (5-hdroxy-1, 4-naphtoquinone) is a natural compound extracted from walnut leaf. (**B**) PiB1 (diethyl-1,3,6,8-tetrahydro-1,3,6,8-tetraoxobenzo[lmn]-phenanthroline-2,7-diacetate) is a chemical synthetic agent.

Pin1 activity can be inhibited by the most commonly used molecule juglone (5-hydroxy-1,4-naphtoquinone), a natural compound extracted from a walnut leaf with irreversible inhibitory action (Figure 1) [25]. A more selective Pin1 inhibitor named PiB (diethyl-1,3,6,8-tetrahydro-1,3,6,8-tetraoxobenzo[lmn]-phenanthroline-2,7-diacetate) (Figure 1) was described by Uchida et al. [26]. Although PiB is a more selective inhibitor, it appears that juglone is more potent as it acts at lower molar concentrations. The aim of this study was to investigate the role of Pin1 in neutrophil functions such as chemotaxis, degranulation and ROS production by using juglone and PiB.

## 2. Materials and Methods

### 2.1. Chemicals and Reagents

Juglone, PiB, cytochalasin B, Bovine alpha Chymotrypsin, Ortho-dianisidine dihydrochloride, LiCl, fMLP, Cytochrome C, PMA, protease and phosphatase inhibitors, buffers, and salt solutions were purchased from Sigma-Aldrich (Saint Quentin Fallavier, France). Polymorphprep is purchased from Axis-Shields (Oslo, Norway). Anti-MPO and anti-NGAL antibodies were from Abcam (Cambridge, UK). HRP-conjugated goat anti-rabbit, HRP-conjugated goat anti-mouse, AP-conjugated goat anti-rabbit antibodies, and luminol chemiluminescence reagents were from Santa Cruz Biotechnology Inc. (Heidelberg, Germany). Dextran T500 was from Pharmacosmos (Holbaek, Denmark). Ficoll was from GE Healthcare Bio-Sciences. SDS–PAGE and Western blotting reagents were purchased from Bio-Rad Laboratories (Hercules, CA, USA). Pin1 peptide substrates were purchased from Bachem (Heidelberg, Germany).

### 2.2. Ethics Statement and Human Neutrophils Isolation

Venous blood was obtained from healthy volunteers after written informed consent had been obtained. The study was approved by the institutional review boards (IRBs) and ethics committee of INSERM (EFS convention number: 2018010827). All these procedures were conducted in accordance with the 1975 declaration of Helsinki, as revised in 2013. Fresh neutrophils were isolated from venous heparinized blood using Polymorphprep gradient centrifugation [21,27]. The neutrophil band was collected, and the cells were washed in PBS and counted. Neutrophils were 96% pure and 99% viable.

### 2.3. Recombinant Pin1 Expression

The *E. coli* BL21 strain carrying the pGEX-plasmid was grown at 37 °C to late stationary phase in Lauria Broth media (20 g/L) containing ampicillin (100 μg/mL) [21,28]. Induction of Pin1 expression was done in the presence of 0.2 mM of isopropyl-thiogalactoside (IPTG) for 3 h at 30 °C. To purify the recombinant protein, cells were harvested by centrifugation (4000× *g*, 4 °C, 20 min). The pellet was resuspended in lysis buffer (50 mM Tris-HCl, 50 mM NaCl, 5 mM MgCl2, 1 mM dithiothreitol (DTT) and protease inhibitors, pH 7.5), after sonication 8 × 30 s on ice the lysate was centrifuged to remove the cellular debris in a Beckman TL100 ultracentrifuge (20 min, 4 °C, 10,000× *g*), GST-recombinant protein was purified with glutathione-Sepharose 4B beads (from Pharmacia) and recombinant protein Pin1 was purified by GST cleavage with thrombin. The recombinant proteins were dialyzed and stored at −80 °C until use.

### 2.4. Pin1 Activity Assay

Pin1 activity was measured using a previously described technique by Fisheret al. (1984) [12] with modifications [21,24]. The assay mixture consisted of 70 µL of buffer (100 mM NaCl, 2 mM DTT, 0.04 mg/mL bovine serum albumin, pH 7.3), 5 µL of purified recombinant Pin1, 5 µL of chemotrypsin (60 mg/mL in 0.001 N HCl). The reaction was started by adding 5 µL of 100 mg/mL of substrate peptide prepared in 480 mM of LiCl and trifluoroethanol and p-nitroaniline. For the cells Pin1, neutrophils at 107 cells/500 µL were suspended and lysed in an ice-cold lysis buffer (50 mM HEPES pH 7.5, 0.25% CHAPS, 100 mM NaCl, 1 mM beta-glycerophosphate, 5 mM NaF and 1 mM EGTA), by using insulin syringe pressure and sonication for 10 s at 4 °C. The assay mixture contains 93 μL HEPES buffer (50 mM HEPES (pH 7.8), 25 μL (60 mg/mL) chymotrypsin solution (Sigma-Aldrich), 6 μL (6 mM) of the peptide Suc-Ala-Glu-Pro-phe-pNA (BACHEM), and 50 μL cell lysate (106 cell equivalent). The absorbance change due to pNA release was followed at 390 nm for 4 min at 10 °C, on the UV-VIS CARY3500 spectrophotometer (Agilent Technologies, Les Ulis, France), results were expressed as OD/min/1 million cells.

### 2.5. Under Agarose Migration Assay

The migration of neutrophils is assessed by the under-agarose migration assay [29,30]. Briefly, 0.7% agarose (Sigma-Aldrich) was dissolved in HBSS by heating for one minute in a microwave. After cooling to 48 °C, 10% of de-complemented FCS was added and 5 mL of the mixture was added to each culture plate (5 cm diameter, falcon plastics). Once the agarose is solidified, four series of three aligned wells (3 mm internal diameter, 3 mm interspace) have been punched in the agarose gel in each plate. The agarose cores are removed with a pipet using a vacuum. For the chemotaxis assay, cells are resuspended to 100 × 10^6^ cells/mL. Cells were treated with increased concentration of Pin1 inhibitors (juglone and PiB) and are added in the central well (5 µL), 5 µL of the chemoattractant fMLP (10^−7^ M) or PBS are placed in the other wells. Finally, the plates are incubated for 2 h at 37 °C. Results were observed using ZEISS inverted microscope. The chemotaxis index is determined as the ratio of chemotaxis/spontaneous migration, results are represented as the ratio/control.

### 2.6. Neutrophil Degranulation Assay

Our approach consists of measuring the extracellular release of granule enzymes such as myeloperoxidase after neutrophils stimulation with fMLP. The MPO activity was measured as previously described [30,31]. Neutrophils (5 × 10^6^ /mL in 500 µL HBSS) were pre-incubated with or without increasing concentrations of Juglone (0, 10, 20, 30 µM) or PiB (0, 25, 50, 75 µM) for 30 min, and 5 µg/mL of cytochalasin B was added 5 min before stimulating with fMLP (10^−6^ M) for 2 min at 37 °C because cytochalasin B is required for azurophil granule release [32,33,34]. Treated cells were centrifuged 30 s at 13,000 rpm (Eppendorf Centrifuge 5415 D, Hamburg, Germany), supernatants were centrifuged again at 13,000× *g* rpm for 10 min at 4 °C and the second supernatants were collected. For the MPO assay, 50 µL of supernatants were mixed with phosphate buffer (350 µL), 50 µL of ortho-dianisidine dihydrochloride (1 mg/mL), and 50 µL hydrogen peroxide (0.0005%). The change in absorbance at 460 nm was recorded by the UV-VIS CARY3500 spectrophotometer (Agilent Technologies), at 22 °C for 10 min. The results were expressed in percentage of control. The supernatants were mixed with 5× concentrated Laemmli sample buffer [35] and denatured for 5 min at 100 °C. Samples were stored at −80 °C until analysis by SDS-PAGE and western blots [36].

### 2.7. Total ROS Production Assay

ROS production was measured by the luminol-enhanced chemiluminescence method [37]. Neutrophils (5 × 10^5^ /500 µL of HBSS) were pretreated or not with increasing concentrations of PiB (0, 25, 50, 75 µM) for 25 min at 37 °C, luminol (10 μM), then pre-activated with TNFα before stimulation with fMLP (10^−7^ M). Chemiluminescence was evaluated with a luminometer (Auto Lumat LB953 model, EG & G Berthold). Light emission was recorded in counted photons per minute (cpm), chemiluminescence was measured for 30 min at 37 °C.

### 2.8. Measurement of Superoxide Production by the Cytochrome C Reduction Assay

Neutrophils (1 × 10^6^ cells) were pre-incubated with TNFα for 15 min before incubation with cytochrome c (1 mg/mL) and PiB (50 μM), during 10 min at 37 °C prior to stimulation with fMLP (10^−6^ M). Superoxide anion production was determined by measuring the ferric cytochrome c reduction with the UV-VIS CARY3500 spectrophotometer (Agilent Technologies) at 550 nm for 10 min. Superoxide production values were calculated using the molar extinction coefficient of reduced cytochrome c [37].

### 2.9. Western Blotting

Proteins were denatured by adding 100 µL of 5 × concentrated Laemmli sample buffer, containing 5 mmoL/L Na3 VO4, 2.5 mmoL/L p-NPP, 10 mmoL/L NaF, 5 mmoL/L EDTA, 5 mmoL/L EGTA, 20 µg/mL leupeptin, 20 µg/mL pepstatin and 20 µg/mL aprotinin. Samples were vigorously vortexed then denatured for 5 min in boiling water (100 °C) and stored at −80 °C until use. Neutrophil lysates were sonicated and subjected to 13% SDS-PAGE (Equivalent of 1 × 10^6^ cells/well) using standard techniques. The separated proteins were transferred to nitrocellulose, then blocked with 5% milk in Tris-buffered saline containing Tween 20 (TBS-T) for 1 h. After blocking, the membranes were probed with the appropriate antibody, anti-MPO, anti-NGAL (1:10,000) overnight at 4 °C. After washing (3 × 5 min) with TBS-Tween 0.1%, the membranes were incubated with HRP-labeled goat anti-rabbit antibody (1:10,000) or HRP-labeled goat anti-mouse antibody (1:10,000). The protein bands were revealed by using enhanced chemiluminescence (Santa Cruz, Heidelberg, Germany). The immune complexes were visualized on the Amersham imager. Densitometric analysis of bands was performed by Image J software, and the data were normalized to total protein.

### 2.10. Statistical Analysis

All the experimental data are expressed as mean ± S.E.M. The analysis was performed using Graph-Pad Prism version 4.0 for windows, and the averages for different groups were compared by a one-way ANOVA test. *p* < 0.05 was considered significant.

## 3. Results

### 3.1. fMLP Induces an Increase of Pin1 Activity in Human Neutrophils which was Inhibited by Juglone and PiB

**Figure 2:**
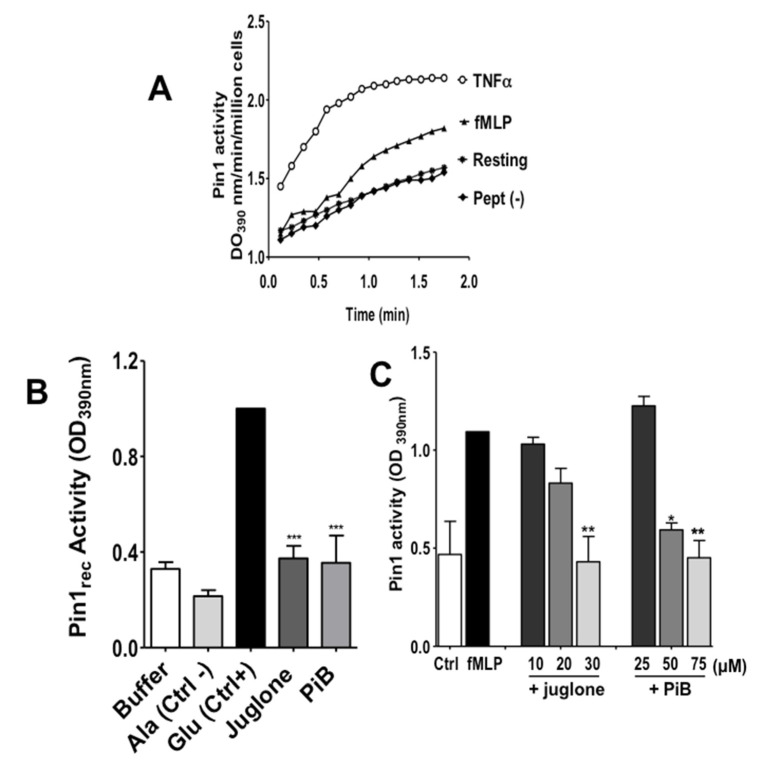
Effect of juglone and PiB on Pin1 activity and its activation in human neutrophils. (**A**) Neutrophils (1 × 10^7^/mL HBSS) were incubated in the absence (Resting) or presence of TNFα (20 ng/mL) for 20 min or fMLP (10^−7^ M) for 1 min. Neutrophils were lysed and Pin1 activity was measured using the Suc-AEPF-p-nitroaniline (pNA) peptide-Glu or Suc-AAPF-p-nitroaniline (pNA) peptide-Ala. The increase of pNA by chemotrypsin was detected at 390 nm. (**B**) Pin1 was expressed and purified from *E. Coli* and was incubated in the absence (Buffer) or presence of juglone or PiB and its activity was assessed using the Suc-AEPF-p-nitroaniline (pNA) peptide-Glu, a Pin1 substrate (Ctrl+) or Suc-AAPF-p-nitroaniline (pNA) peptide-Ala, a negative control peptide (Ctrl−). The increase of OD due to pNA release by chemotrypsin was detected at 390 nm. (**C**) Neutrophils (1 × 10^7^/mL HBSS) were incubated in the absence or presence of different concentrations of juglone or PiB and stimulated by fMLP (10^−7^ M) for 1 min. Neutrophils were lysed and Pin1 activity was measured using the Suc-AEPF-p-nitroaniline (pNA) peptide-Glu. The increase of pNA by chemotrypsin was detected at 390 nm. Ctrl corresponds to resting neutrophils. Data are means ± S.E.M. of 3 separate experiments (* *p* < 0.05, ** *p* < 0.001, *** *p* < 0.0001 juglone and PiB as compared to control).

To investigate the implication of Pin1 in fMLP-induced neutrophil activation, we first wanted to know if Pin1 is activated in fMLP-stimulated neutrophils. Human neutrophils were treated or not by fMLP then lysed. TNFα was used as a positive control as it induced a strong activation in human neutrophils (21). The activity of Pin 1 was determined by measuring the absorbance of free pNA resulted from the cleavage of Suc-Ala-Glu-Pro-Phe-pNA after the *cis* to *trans* conformational changes. Results presented in Figure 2A show that fMLP strongly increased Pin1 activity. Neutrophils are terminally differentiated short-lived cells resistant to transfection. To study the role of specific enzymes such as Pin1 in neutrophils, we used two cell-permeant pharmacological Pin1 inhibitors, juglone and PiB (25, 26) (Figure 1). We checked that juglone and PiB were able to inhibit recombinant Pin1 activity in vitro at 20 µM and 50 µM respectively (Figure 2B). Interestingly, treatment of neutrophils with juglone or PiB, before fMLP stimulation, markedly reduced fMLP-induced Pin1 activity with a concentration-dependent effect (Figure 2C), further showing that this assay is specific for Pin1. These results clearly show that the bacterial peptide N-formyl-methionyl-leucyl-phenylalanine (fMLP), a G-protein coupled receptor (GPCR) agonist-induced Pin1 activation in human neutrophils.

### 3.2. Juglone and PiB Inhibit Neutrophil Chemotaxis

**Figure 3:**
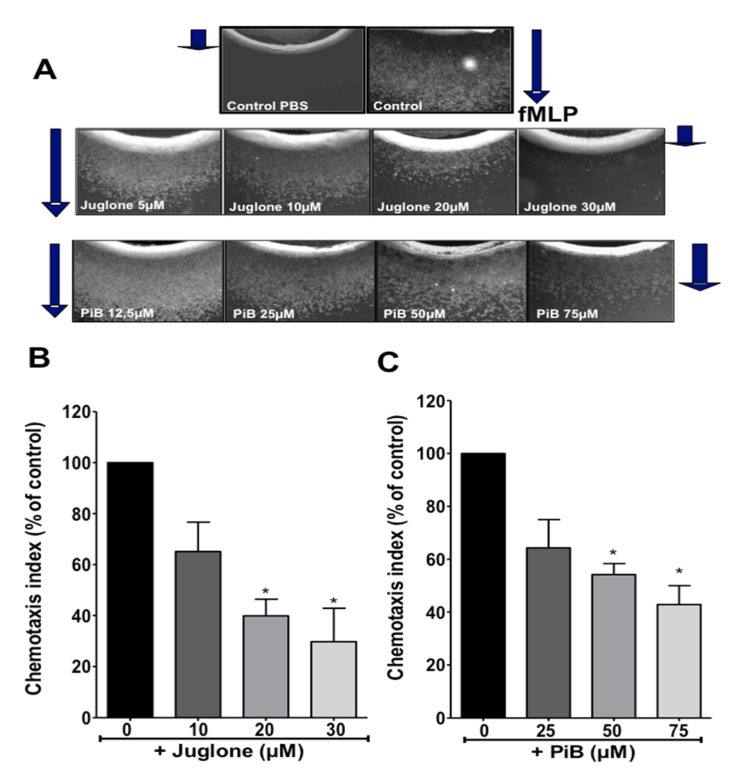
Effect of juglone and PiB on neutrophil chemotaxis. (**A**) Neutrophils (100 × 10^6^ cell/mL) were pretreated with increasing concentration of juglone (0, 10, 20, 30 µM) or PiB (0, 25, 50, 75 µM). Cells (5 µL) are added to the agarose central well, 5 µL of the chemoattractant fMLP (10^−7^ M) or buffer PBS are placed in the other wells, the plates are incubated for 2 h at 37 °C. Results were observed using a ZEISS microscope. The direction of migration towards fMLP is shown by the arrows. (**B**,**C**) The chemotaxis index is determined as the distance of cell migration to fMLP. Results are represented as the ratio to fMLP without inhibitors (100%). Histograms represent the percentage of inhibition of cell migration, in the presence of Pin1 inhibitors. Data are means ± S.E.M. of 4 separate experiments (* *p* < 0.01).

Chemotaxis towards the gradient of chemoattractants is fundamental for neutrophil recruitment to the inflammatory sites. Here, we used the under-agarose chemotaxis technique to test the role of Pin1 in this key neutrophil function. As shown in Figure 3A (upper images), in the presence of PBS in the opposite well, neutrophils did not move out of the well, however, in the presence of fMLP, neutrophils extensively moved to the well-containing fMLP. Incubation of neutrophils in the presence of increasing concentrations of juglone (0, 10, 20, 30 µM) or PiB (0, 25, 50, 75 µM) resulted in inhibition of neutrophil migration. Quantification of chemotaxis index of several experiments showed that juglone and PiB decreased significantly neutrophil chemotaxis in a dose-dependent manner (Figure 3B,C). The inhibitory effect was more pronounced with juglone-treated cells, the chemotaxis was completely inhibited at 30 μM for some donors. Juglone and PiB did not affect cell viability even at the highest concentration used (data not shown). These results suggest that Pin1 controls fMLP-induced neutrophil chemotaxis and recruitment to the inflammatory site.

### 3.3. Juglone and PiB Inhibit fMLP-Induced Neutrophil Degranulation of Azurophil and Specific Granules

**Figure 4:**
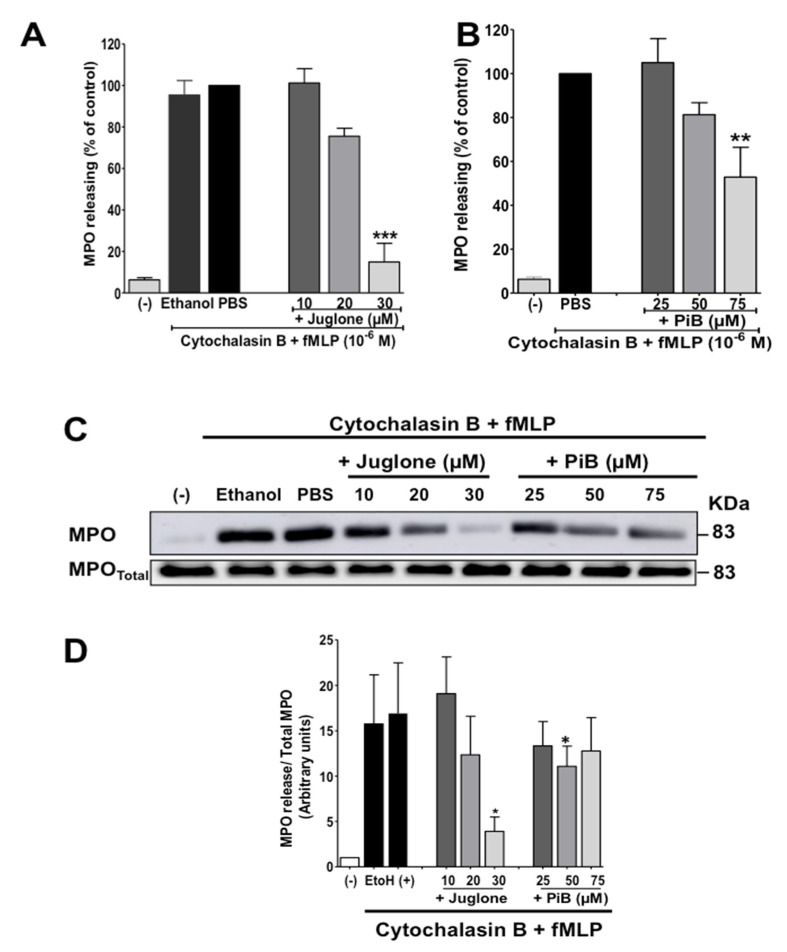
Effect of juglone and PiB on MPO release by human neutrophils. (**A**,**B**) Cells were pretreated or not with increasing concentration of Juglone dissolved in ethanol (0, 10, 20, 30 µM) and PiB (0, 25, 50, 75 µM), then with cytochalasin B (5 µg/mL) for 5 min before stimulation with fMLP (10^−6^ M). Control cells were treated with ethanol (0.001% or PBS), then cytochalasin B for 5 min and stimulated by fMLP in the same conditions. After centrifugation the supernatant is recovered to measure MPO activity using the spectrophotometric method as reported in the methods section. (**C**) Equal amounts of supernatant samples and neutrophil pellets were subjected to SDS-PAGE followed by immunoblot analysis with anti-MPO antibody. A representative Western blot of 4 experiments is shown. (**D**) Western blot results were quantified and expressed in arbitrary units (AU). Values are means ± S.E.M. *n* = 4 (* *p* < 0.05, ** *p* < 0.01, *** *p* < 0.001).

Neutrophil degranulation is an essential function required for innate immunity and inflammation. We then tested the effect of juglone and PiB on fMLP-induced neutrophil degranulation of azurophil and specific granules using myeloperoxidase (MPO) and NGAL/Lipocalin2 as markers respectively. Neutrophils were first incubated without or with juglone or PiB, second incubated with cytochalasin B, as this agent is required for degranulation, then stimulated by fMLP. MPO was first detected by measuring its activity and then confirmed by detecting it by Western Blot using a specific antibody. Results show that fMLP in the presence of cytochalasin B, induced release of MPO in the extracellular media, and that juglone and PiB inhibited this release as measured by its enzymatic activity (Figure 4A,B). The ethanol control at 0.001% (used to dissolve inhibitors) did not affect neutrophil degranulation. To rule out the possibility that juglone and PiB could interfere with the enzymatic assay, we further performed Western Blot analysis. The results show that effectively, juglone and PiB inhibited fMLP-induced MPO release from azurophil granules (Figure 4C,D).

**Figure 5:**
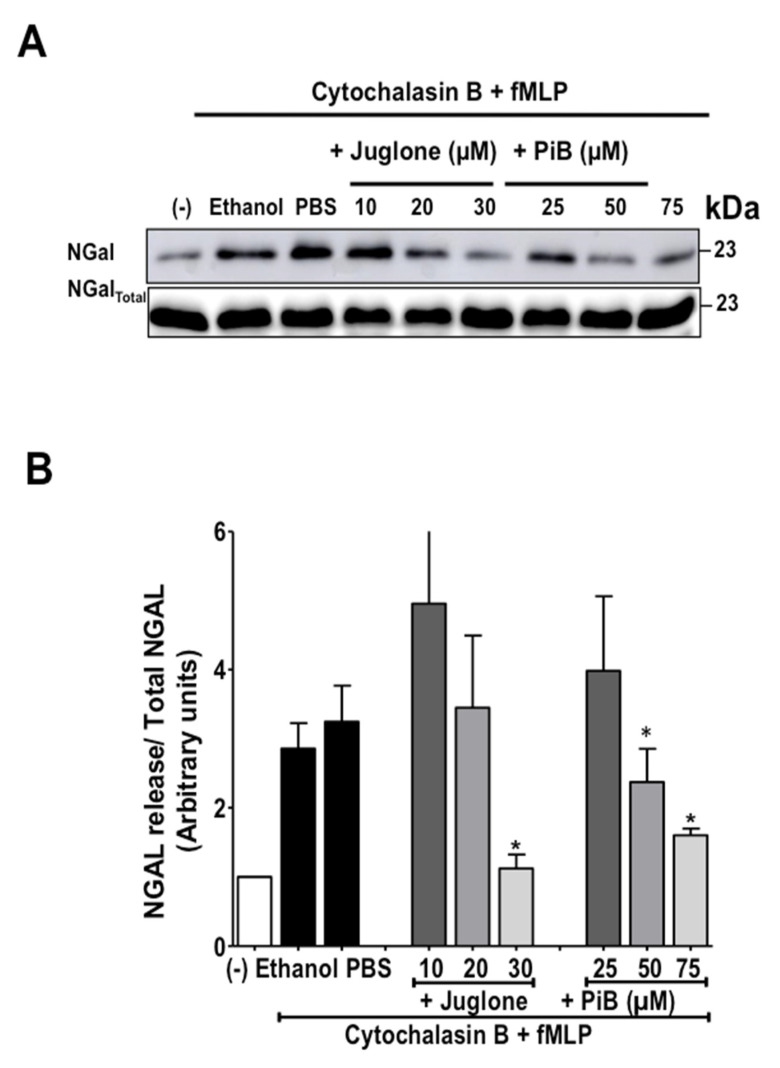
Effect of juglone and PiB on NGal release by human neutrophils. (**A**) Cells were pretreated or not with increasing concentration of Juglone dissolved in ethanol (0, 10, 20, 30 µM) and PiB (0, 25, 50, 75 µM), then with cytochalasin B (5 µg/mL) before stimulation with fMLP (10^−6^ M). Control cells were treated with ethanol (0.001% or PBS) then with Cytochalasin B (5 µg/mL) and stimulated by fMLP in the same conditions. After centrifugation, equal amounts of supernatant samples and neutrophils lysates were subjected to SDS-PAGE followed by immunoblot analysis with anti-NGal antibody. A representative Western blot of 4 experiments is shown. (**B**) Western blot results were quantified and expressed in arbitrary units (AU). Values are means ± S.E.M. *n* = 4 (* *p* < 0.05).

In order to address the question if Pin1 is also involved in fMLP-induced specific granule release, we studied the release of NGAL a marker of specific granules detecting it by Western Blot using a specific antibody. Results show that in combination with cytochalasin B, fMLP induced release of NGAL in the extracellular media and that juglone and PiB inhibited NGAL protein release from specific granules (Figure 5). These data corroborate with the previous results and suggest that Pin1 controls fMLP-induced neutrophil degranulation of specific granules. These results suggest that Pin1 controls neutrophil degranulation.

### 3.4. PiB Decreased Both Total ROS and Superoxide Anion Production

**Figure 6:**
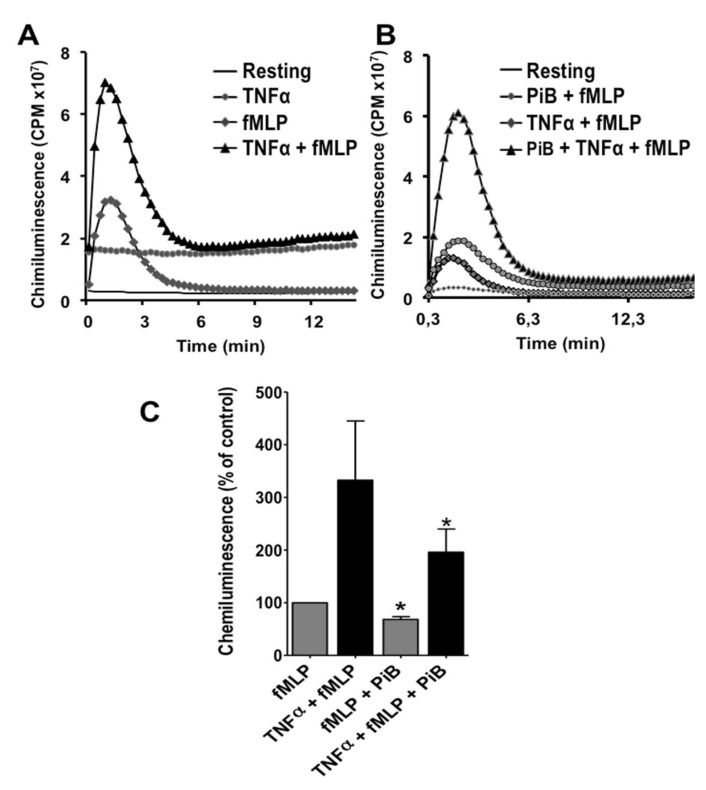
Effect of PiB on ROS production by human neutrophils. (**A**) Neutrophils (5 × 10^5^ cells/0.5 mL) were incubated or not with TNFα (20 ng/mL) for 20 min before stimulation with fMLP (10^−7^ M). ROS productions were measured by luminol-enhanced chemiluminecence assay. This result represents an example of ROS production of resting or in the presence of TNFα, fMLP or TNFα + fMLP. (**B**) Neutrophils (5 × 10^5^ cells/0.5 mL) were incubated or not with PiB (50 µM) for 30 min, then treated or not with TNFα (20 ng/mL) for 20 min before stimulation with fMLP (10^−7^ M). ROS productions were measured with luminol-enhanced chemiluminecence assay. (**C**) Histograms representing ROS production as calculated by the mean of the total area under the chemiluminescence curves. Data are means ± S.E.M. of 3 separate experiments (* *p* < 0.05).

We have previously shown that juglone inhibited NADPH oxidase priming [21,22,23,24]. Here we evaluated the effect of PiB on fMLP-induced ROS production and TNF-induced priming. We measured ROS production by the luminol-enhanced chemiluminescence assay which detects multiple forms of ROS, mainly superoxide anion, hydrogen peroxide and hypochlorous acid. Results show that fMLP (at 10^−7^ M) induced release of low level of ROS, whereas pre-incubation of cells with TNFα (20 ng/mL) enhanced the fMLP-induced response (Figure 6A). PiB (at 50 µM) decreased significantly fMLP-induced ROS production and TNF-primed response (Figure 6B,C).

**Figure 7:**
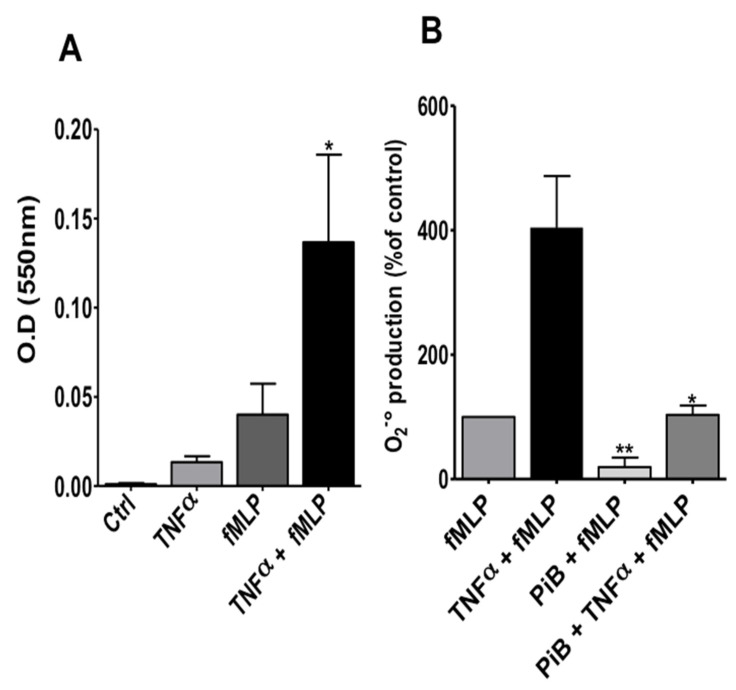
Effect of PiB on superoxide anion production by human neutrophils. (**A**) Neutrophils (1 × 10^6^/mL HBSS) were incubated in the absence or presence of TNFα (20 ng/mL) for 20 min before stimulation with fMLP (10^−7^ M). Superoxide production was determined by measuring ferricytochrome c reduction at 550 nm over 10 min at 37 °C. (**B**) Neutrophils (1 × 10^6^/mL HBSS) were incubated without or with PiB (50 µM) for 30 min, then treated or not with TNFα (20 ng/mL) for 20 min before stimulation with fMLP (10^−7^ M) and superoxide production was determined by measuring ferricytochrome c reduction at 550 nm over 10 min at 37 °C. Data are means ± S.E.M. of 3 separate experiments (* *p* < 0.05; ** *p* < 0.01).

Luminol-amplified chemiluminescence is a very sensitive technique which reflects several processes such as NADPH oxidase activation, degranulation, MPO activity and availability of ROS molecules. In order to investigate the effect of PiB on NADPH oxidase activation, we evaluated its effect on superoxide anion production, using cytochrome c reduction assay, a specific technique which reflects more precisely NADPH oxidase activation. As shown in Figure 7A, fMLP alone induced a low superoxide production, whereas incubation with TNFα before fMLP stimulation, resulted in more superoxide production. Figure 7B shows that PiB significantly inhibited superoxide anion release by fMLP-stimulated and TNF-primed neutrophils. These results suggest that Pin1 could control the fMLP-induced activation of NADPH oxidase in human neutrophils.

## 4. Discussion

The present study aims to investigate the role of the peptidylprolylcis/trans isomerase Pin1 in neutrophil key functions, i.e., chemotaxis, degranulation and superoxide anion production. Here we show that the chemotactic peptide fMLP induced Pin1 activation in human neutrophils. Using two different Pin1 inhibitors, juglone and PiB we showed that they are able to dramatically decrease neutrophil chemotaxis, degranulation and superoxide production. These results suggest that Pin1 triggers neutrophil functions required for innate immunity and inflammation and these new data constitute an important addition to the understanding of neutrophil activation.

FMLP acts on neutrophils via a specific receptor called FPR (formylated peptide receptor). This receptor belongs to the G-protein coupled receptor (GPCR) family. Our results show that in addition to cytokine and TLR receptors [21,22,23,24], FPR are also able to activate Pin1 in cells. The pathways involved in Pin1 activation by these agonists in neutrophils are not known and are under investigation.

Neutrophils are terminally differentiated and short-lived cells resistant to transfection. An alternative strategy to study the role of specific enzymes is to use cell-permeant pharmacologic inhibitors. In this study we used juglone and PiB, which are known Pin1 inhibitors [25,26], to analyze the role of this enzyme in neutrophil chemotaxis, degranulation and superoxide anion production. Juglone is a natural compound purified from nuts leaves, which was found to inhibit Pin1 activity but also have other effects such as inhibition of RNA polymerase I, II and III [38] and inactivation of other cysteine-rich proteins required for cell cycle progression [39]. However, PiB is chemically synthetized and inhibits Pin1 activity, at our knowledge, no other effects were described up to date [26]. Obtaining the same result with both inhibitors, strongly supports the role of Pin1 in neutrophil functions. However, other targets of juglone and PiB could explain the observed effects.

The oriented migration of neutrophils towards the infection site is an important step in the inflammatory response. Neutrophil chemoattractants include the bacterial peptide fMLP, IL-8, C5a and leukotriene B4 (LTB4) [3,4]. Here we showed that PiB and juglone significantly decreased neutrophil under agarose chemotaxis in vitro. Pin1 was reported to control Epstein-Barr virus-induced gene 2 (EBI2)-induced eosinophil migrations [40]. Chemotaxis is known to be dependent on AKT and p38MAPKinase [41,42]. P38MAPKinase can phosphorylate proteins on “-Ser/Thr-Pro-” sites which are Pin1 substrates. These proteins substrates of p38MAPKinase and Pin1 involved in neutrophil migration remain to be identified.

The release of granules contents is also a key neutrophil’s function. PiB and juglone significantly decreased fMLP-induced MPO and N-Gal release from azurophil and specific granules respectively in combination with cytochalasin B. These results were clearly confirmed by two approaches, MPO-enzymatic activity and by western blotting. fMLP induces several signaling pathways, such as the PI3K–AKT pathway, the Rac–p38MAPK pathway, and the Ras–ERK1/2 pathway [31,32]. MEK1-ERK1/2 and p38MAPkinase pathways are associated with the neutrophil’s inflammatory response and are involved in the chemotaxis and degranulation [41,42]. These MAPKinases and other proline-directed kinases could phosphorylate several proteins on a “Ser/Thr-Pro” sequence that can be a substrate for Pin1. Identification of these phosphorylated proteins is important to understand how Pin1 controls these functions. TNF-induced neutrophil degranulation was shown to be independent of Pin1, as juglone was without effect [43]. These data suggest that the involvement of Pin1 in neutrophil degranulation is agonist dependent.

In previous studies, using juglone, we showed that Pin1 is required for priming of ROS production induced by TNFα [21], CL097 [22], and LPS [24] and recently for fMLP-induced ROS production [24]. PiB, another selective inhibitor of Pin1 confirmed the results obtained with juglone. The prolyl-isomerase Pin1 is known to bind to phosphorylated Ser345 of p47phox and to change its conformation to facilitate its phosphorylation by PKC and its binding to p22phox and NADPH oxidase assembly and activation [21]. The result obtained with PiB confirmed our previous result obtained using juglone, providing more evidence for the involvement of Pin1 in TNFα-induced NADPH oxidase priming and activation.

In summary, in this study we show that fMLP, a GPCR mediated agonist-induced the activation of the prolyl-isomerase Pin1 in human neutrophils, Pin1 inhibitors impair pro-inflammatory functions of neutrophils such as migration, degranulation, and superoxide and ROS production elicited by GPCR activation and suggest that Pin1 could control neutrophil inflammatory functions.

## Data Availability

Not applicable.
